# Genotypic and Environmental Effects on Morpho-Physiological and Agronomic Performances of a Tomato Diversity Panel in Relation to Nitrogen and Water Stress Under Organic Farming

**DOI:** 10.3389/fpls.2022.936596

**Published:** 2022-06-29

**Authors:** Pasquale Tripodi, Maria R. Figàs, Fabrizio Leteo, Salvador Soler, María José Díez, Gabriele Campanelli, Teodoro Cardi, Jaime Prohens

**Affiliations:** ^1^CREA Research Centre for Vegetable and Ornamental Crops, Pontecagnano, Italy; ^2^Instituto de Conservación y Mejora de la Agrodiversidad Valenciana, Universitat Politècnica de València, Valencia, Spain; ^3^CREA Research Centre for Vegetable and Ornamental Crops, Monsampolo del Tronto, Italy

**Keywords:** tomato, local varieties, nitrogen stress, water stress, sustainability, organic farming, genotype × environment interaction, breeding

## Abstract

The agricultural scenario of the upcoming decades will face major challenges for the increased and sustainable agricultural production and the optimization of the efficiency of water and fertilizer inputs. Considering the current and foreseen water scarcity in several marginal and arid areas and the need for a more sustainable farming production, the selection and development of cultivars suitable to grow under low-input conditions is an urgent need. In this study, we assayed 42 tomato genotypes for thirty-two morpho-physiological and agronomic traits related to plant, fruit, and root characteristics under standard (control) and no-nitrogen fertilization or water deficit (30% of the amount given to non-stressed trials) treatments in two sites (environments), which corresponded to organic farms located in Italy and Spain. A broad range of variation was found for all traits, with significant differences between the applied treatments and the cultivation sites. Dissection of genotypic (G), environmental (E), and treatment (T) factors revealed that the three main factors were highly significant for many traits, although G was the main source of variation in most cases. G × E interactions were also important, while G × T and E × T were less relevant. Only fruit weight and blossom end rot were highly significant for the triple interaction (G × E × T). Reduction of water supply significantly increased the soluble solid content in both locations, whereas both nitrogen and water stress led to a general decrease in fruit weight and total yield. Despite so, several accessions exhibited better performances than the control when cultivated under stress. Among the accessions evaluated, hybrids were promising in terms of yield performance, while overall landraces and heirlooms exhibited a better quality. This suggests the possibility of exploiting both the variation within ancient varieties and the heterosis for yield of hybrids to select and breed new varieties with better adaptation to organic farming conditions, both under optimal and suboptimal conditions. The results shed light on the strategies to develop novel varieties for organic farming, giving hints into the management of inputs to adopt for a more sustainable tomato cultivation.

## Introduction

Water and nitrogen (N) are major inputs in agriculture, representing key elements to ensure the high productivity and quality of crops. It is estimated that plants globally consume over 70% of the freshwater from surface or groundwater resources (blue footprint) (Hoekstra et al., 2012) and up to 99% considering the volume of rainwater absorbed during the grown cycle (green footprint) (Mekonnen and Hoekstra, 2014). The availability of water supply in many areas of the world is threatened by future demographic trends and climate change (Kummu et al., 2012; Rodell et al., 2018). Indeed, the global population is expected to reach over 9 billion in the coming few decades, and this will increase the competition between agriculture and other sectors (e.g., industrialization, urbanization) for the need of resources (Tilman et al., 2011; Smith, 2013). Moreover, the rise of temperature could result in a greater demand and scarcity of water resources, leading to an increase in the frequency of drought stress. Drought combined with the intensive exploitation of groundwater that lowers the aquifer table increases the soil salinity and desertification (Heggy, 2018) and represents the most devastating threat to crop yield particularly in marginal and arid areas (Ding et al., 2018; Du et al., 2018). In this scenario, soil infertility is a major risk for agriculture, considering that almost a third of the world’s productive lands has been already degraded (Wall and Six, 2015) with an estimated annual increase of 12 million hectares at risk of losing fertility which affects over 1.5 billion people globally (UNCCD, 2020).

In plant nutrition, N is an important component of many organic structural compounds and secondary metabolites (Zrenner et al., 2006), playing a fundamental role in photosynthesis and regulating the uptake of water and nutrient (Bian et al., 2020). Industrial agriculture has tremendously raised the use of fertilizers, leading to a global amount of N from fertilizers equal to that naturally fixed from the atmosphere (Gojon, 2017). The excess of N not used by crops is subjected to denitrification being converted by soil organisms, mostly bacteria, in gaseous forms (e.g., nitrous oxide and dinitrogen gas) that escape into the atmosphere, contributing to the rise of global warming (Maximillian et al., 2019). In addition, the fraction of N leached from the soil to groundwater contributes to nitrate pollution, which causes adverse effects to the environment and human health (Huan et al., 2020; Yu et al., 2020).

Therefore, the future risks for the entire ecosystem linked to the uncontrolled use of resources posed the basis for new plans aiming at a more sustainable and environmentally friendly agriculture (Jhariya et al., 2019). However, the achievement of such progresses needs to consider different factors, including the type of crop, the variation due to genetic and environmental conditions, as well as the agronomic management practices (The et al., 2021).

Tomato (*Solanum lycopersicum*) is a highly demanding vegetable in terms of N and water supplies (Zotarelli et al., 2009; Ayankojo et al., 2020) with an estimated uptake ranging from 200 to 400 kg N/ha based on pedological conditions (Zhang et al., 2011; Cheng et al., 2021), and a daily uptake of up to 1.8 L plant^–1^, depending on solar radiation and temperature (Hanping et al., 2017). In the past two decades, the global production grew from 109⋅10^6^ tons in 2,000 to 186⋅10^6^ tons in 2020 (FAOSTAT, 2021), on a total surface that increased by 31% in the same period, up to 5 million hectares, which represents, to date, the largest world cultivation area among vegetables. Such a trend that required increasingly higher inputs that combined with the low cost of chemical fertilizers has generally led to over N fertilization. Despite the fact that N supply is beneficial to achieve higher production in crops, it has been reported that excessive N fertilization results to detrimental effects on quality and yield (Elhanafi et al., 2019; Ben Mariem et al., 2020). In the case of tomato, it has been observed that beyond certain levels, N has no beneficial effect on the overall performance of the crop (Truffault et al., 2019; Hernández et al., 2020). The reduction of yield due to N and H_2_O deficit is related to several factors including cultivation techniques, varieties, soil types, and the phenological phase in which the stress occurs. A yield reduction ranging from 15 to 37% when water input decrease to 50–60% ET has been reported (Lovelli et al., 2017; Du et al., 2018). Thus, it is necessary to define a rational use of resources during the plant life cycle, optimizing the irrigation and fertilization management, to secure an adequate food level with less water and/or N supply. This goal can be achieved through the adoption of sustainable agricultural production such as organic farming systems and the selection of cultivars able to maximize the water- and N-use efficiency. It must be considered that breeding programs, in general, are performed under ideal N and water supply conditions, resulting in cultivars not performing well in stress conditions (van Bueren and Struik, 2017). Instead, exploring the existing panel of resilient local varieties selected in harsh environments can represent a viable strategy to develop new materials suitable for low-input cultivation conditions (Fullana-Pericàs et al., 2019; Rosa-Martínez et al., 2021).

Organic agriculture is a sustainable and environmentally friendly strategy based on the reduction of chemical inputs replaced by fertilizer and pesticides from organic sources (e.g., plant and microbial extracts) (Vogt, 2007). Globally, there are a total of 72.2 million hectares of organic or in-conversion agricultural surface, with Europe being the second continent in terms of area invested and Spain and Italy among the most representative countries (FIBL and IFOAM, 2021). The European Green Deal Strategy has set the objective of reaching 25% of the EU agricultural area under organic conditions before 2030. Therefore, continuous efforts to achieve such a target by the end of the decade are required. Since organic farming systems do not rely on the intensive use of external means, it is necessary that plants are able to self-regulate and cope with hostile crop conditions. Such self-regulation systems imply that interactions between genotype and environment play a key role in organic systems.

It is estimated that yields under organic systems are typically lower than in conventional ones. Meta-analyses highlighted a reduction of 20–25% in organic farms (de Ponti et al., 2012; Seufert et al., 2012; Gong et al., 2022), although this depends on several factors including the management practices, rotation adopted, and variety choice (Li et al., 2019), thus suggesting the need to identify suitable genotypes for organic cultivation considering different cultivation practices. In this study, 42 tomato accessions were evaluated for morpho-physiological parameters and agronomic performances in organic conditions under no-N fertilization and reduced water supply conditions and compared to a control with standard supplies of both inputs. The panel set represented different varietal groups including landraces and cultivars retrieved from different areas and pre-selected for diversity and good performance under organic cultivation conditions (Tripodi et al., 2021). We considered Italy and Spain as representative areas of the Mediterranean basin for cultivation under organic farming, being among the first nine world countries with the largest area of organic agricultural land (Willer and Lernoud, 2019) and considering those areas at risk of extensive drought periods. The objectives of this work were to (i) provide new information on tomato germplasm resilient to drought and low N input; (ii) explore the effects of genotype, environment, and water or N stress treatments for selecting the best materials for organic tomato cultivation; (iii) determine how the applied stresses affect the plant, root, and fruit traits. Furthermore, the approach pursued gives hints into the strategies to adopt in terms of management of irrigation and N application in tomato grown in organic conditions.

## Materials and Methods

### Plant Material

**FIGURE 1:**
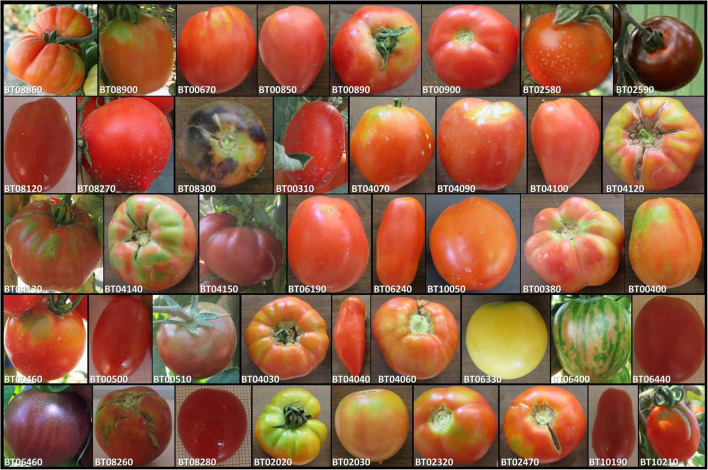
Fruit morphological diversity of the 42 accessions evaluated in two organic farms under nitrogen and water stress conditions in this study. BL = Breeding lines, CL = Elite cultivars, FM = Landraces for the fresh market, HL = Heirlooms, LS = Long shelf-life cultivars. Additional details in are Supplementary Table 1. The pictures are not in the same scale.

The plant material consisted of 42 cultivated tomato (*S. lycopersicum*) genotypes belonging to diverse cultivar groups including elite cultivars (CL, 9) and heirlooms (HL, 6) with different origins, landraces for the fresh market (FM, 21) from Spain and Italy, and long shelf-life cultivars “de penjar/da serbo” (LSL, 6) originating from different regions in the Spanish eastern coast and Southern Italy. The panel set was assembled from the germplasm collections of the Research Centre for Vegetable and Ornamental Crops (CREA; Italy) and the Universitat Politècnica de València (UPV; Spain) and selected from a core collection previously assayed (Tripodi et al., 2021). Two additional breeding lines (BL, 2) developed from ongoing breeding programs were included. The morphological fruit diversity of the collection studied is shown in Figure 1. Germplasm details are in Supplementary Table 1.

### Experimental Field Trials

The plants were grown during the spring-summer of 2020 in two fields certified for organic farming. The first field was the experimental station of the Research Centre for Vegetable and Ornamental Crops (CREA, Monsampolo del Tronto, AP, Italy, hereafter IT) (42°53′ N, 13°48′ E, 40 m.a.s.l), and the second was a private farm of the municipality of Alcàsser (province of Valencia, Spain, hereafter SP) (39°23′ N, 0°27′ W, 29 m.a.s.l). The temperature and humidity during the growth season as well as the soil chemical properties determined before the start of the experiments at each site are reported in Table 1. According to the typical tomato cycles of the two growing areas, plants were sown at the end of February (SP) and at the beginning of April (IT) and transplanted to the open field after 6 weeks. In Spain, after the transplant, the plants were covered with a textile film using the microtunnel technique to protect from low temperatures. This cover was maintained until the second week of April. The number of days of the culture (from planting to the end of the crop) was 103 and 95 days in IT and SP, respectively. In both fields, along with the control, N- or water-stress experimental conditions were applied. The fertilization in the control plot in IT consisted of a dose of 205 kg N ha^–1^, 94 kg ha^–1^ P_2_O_5_, and 44 kg ha^–1^ K_2_O, while in Spain, it consisted of 249 kg N ha^–1^, 72 kg ha^–1^ P_2_O_5_, and 72 kg ha^–1^ K_2_O. In IT, basic fertilization with “Superstallatico” organic pellets (Unimer, Milano, Italy) (3-0-0) and “Prodigy4” (7-6-1) (Biogard, Grassobbio, Italy) was applied. The rest of the N and potassium units were supplied in fertigation with the water soluble based on hydrolyzed animal epithelium “Gold Dust” (15-0-0) (K-Adritica, Loreo, Italy) and the water soluble based on crystalline potassium sulfate plus amino acids “Prokton” (6-0-17) (Biogard, Grassobbio, Italy). In SP, the fertilization was supplied with the irrigation system using “Fenorganic Nitrógeno” (16-0-0) (Fenorganic, Archidona, Spain) for N, “Fosfoser” ECO GR (Mapryser, Barcelona, Spain) for P, and “Summum Líquido Quality” (0-0-15) (Fertinagro, Teruel, Spain) for K to provide the desired fertilization levels. All these fertilizers are authorized in organic farming. The N-stress plot (NS) consisted in the absence of N supply (0 kg N ha^–1^).

**TABLE 1:** Pedoclimatic conditions of the two organic fields: (i) the experimental station of the Research Centre for Vegetable and Ornamental Crops (CREA) located in Monsampolo del Tronto, Italy; (ii) a private farm located in Alcàsser, province of Valencia, Spain.

	Soil composition						Temperature °C			Relative humidity (%)		
Location	Organic matter content (%)	Electrical conductivity^a^	Total nitrogen content^b^	Phosphorous^c^	pH	USDA Soil texture class	Max	Min	Average	Max	Min	Average
Italy	1.00	1,018	1.04	70.7	8.0	Loam^d^	34.2	10.4	23.1	96	33	69.9
Spain	1.67	280	1.24	16.3	8.2	Clay-loam^e^	34.4	7.2	21.3	99	18	73

*^a^μS cm^–1^; ^b^g kg^–1^ dm; ^c^mg kg^–1^ dm.*

*^d^20% of Clay (< 0.002 mm), 30% of fine silt (0.002 to 0.02 mm), 30% of silt (0.02 to 0.05 mm), 50% of total sand (0.05 to 2 mm).*

*^e^32.95% of Clay (< 0.002 mm), 14.5% of fine silt (0.002 to 0.02 mm), 15.5% of silt (0.02 to 0.05 mm), 37.05% of total sand (0.05 to 2 mm).*

The total amount of water supplied was based on the calculation of crop evapotranspiration (ETc). In this way, for IT, the irrigation volume was 3,801 m^3^/ha plus a rainfall of 938 m^3^/ha considering a daily global solar radiation of 257 W/m^2^, a mean reference crop evapotranspiration (mean Eto) of 45.8 m^3^/ha, and the total reference crop evapotranspiration (total Eto) of 4,719 m^3^/ha. For SP, the irrigation volume was 3,212 m^3^/ha plus a rainfall of 1,126 m^3^/ha, with a daily global solar radiation of 279 W/m^2^, mean reference crop evapotranspiration (mean Eto) of 46.9 m^3^/ha, and total reference crop evapotranspiration (total Eto) of 4,451 m^3^/ha.

The water stress treatment (WS) target consisted in 30% of water supply with respect to the control, finally resulting in 1,146 m^3^/ha in IT and 937 m^3^/ha in SP. Considering rainfall, these were equal to about 44 and 47% of total water with respect to the controls in IT and SP, respectively. The plants were irrigated throughout the entire cultivation period using a drip irrigation system. At both locations, a randomized block design was followed with three blocks for each treatment (control, WS and NS) for a total of nine blocks. In each block, four plants/accessions were considered for a total of 3,024 plants across the two locations. A biodegradable plastic mulch film was used to avoid competition with weeds. When necessary, weeding was done manually. Plants were grown at a density of 2.6 plant/m^2^ using a system of double rows in which canes from adjacent rows were tied together forming a triangle-shaped structure.

### Morphological and Agronomic Trait Measurements

All plants in each block were individually phenotyped for 9 pseudo-qualitative traits related to plant architecture and fruit characteristics and 16 agronomic traits, including both pseudo-qualitative and quantitative measurements. All traits were scored at both locations except for plant vigor, which was scored only in Spain. The details of traits and scale or method of measurement are listed in Table 2. All data have been manually reviewed and curated prior to analysis.

**TABLE 2:** List of traits analyzed and method of measurement for the 42 cultivated tomato accessions analyzed in control, water deficit, and nitrogen stress conditions in Italy and Spain.

Trait	Acronym	Organ^a^	Type^b^	Scale/method of measurement
**Morphological plant and fruit descriptors**				
Foliage density	FD	PL	PQ	3 = sparse; 5 = intermediate; 7 = dense
Plant vigor*	PV	PL	PQ	1 = very weak; 3 = weak; 5 = medium; 7 = strong; 9 very strong
Style position	SP	PL	PQ	1. inserted; 2. same level as stamen; 3. slightly exerted; 4. highly exerted
Fruit set sequence	FS	FR	PQ	3 = poor; 5 = intermediate; 7 = good; 9 = very good
Green shoulder	GS	FR	PQ	0 = absent; 1 = light green; 2 = medium green; 3 = dark green
Puffiness appearance	PA	FR	PQ	1 = absent; 3 = slight; 5 = intermediate; 7 = severe
Blossom end scar condition	BE	FR	PQ	1 = open; 2 = closed; 3 = both
Fruit firmness	FF	FR	PQ	1 = very soft; 2 = soft; 3 = medium; 4 = hard
Ribbing at calyx end	RC	FR	PQ	1 = very weak; 2 = weak; 3 = intermediate;4 = strong; 5 = very strong
**Agronomic traits**				
Stem diameter	SD	PL	QN	Using a caliper (cm)
Radial cracking	RK	FR	PQ	1 = absent; 3 = scarce (< 5% of fruits affected); 5 = intermediate (5% < 20%); 7 = abundant (> 20%)
Concentric cracking	CC	FR	PQ	1 = absent; 3 = scarce (< 5% of fruits affected); 5 = intermediate (5% < 20%); 7 = abundant (> 20%)
Fruit fasciation	FFA	FR	PQ	1 = absent; 3 = slight (< 5% of fruits affected); 5 = intermediate (5% < 20%); 7 = severe (> 20%)
Blossom-end Rot	BR	FR	PQ	1 = absent; 3 = slight (< 5% of fruits affected); 5 = intermediate (5% < 20%); 7 = severe (> 20%)
Pests in foliage	PF	PL	PQ	1 = very scarce; 3 = scarce; 5 = intermediate; 7 = severe; 9 = very severe
Pests in fruits	PFr	FR	PQ	1 = absent; 3 = scarce (< 5% of fruits affected); 5 = intermediate (5% < 20%); 7 = abundant (> 20%)
Disease in foliage	DF	PL	PQ	1 = very scarce; 3 = scarce; 5 = intermediate; 7 = severe; 9 = very severe
Disease in fruits	DFr	FR	PQ	1 = absent; 3 = scarce (< 5% of fruits affected); 5 = intermediate (5% < 20%); 7 = abundant (> 20%)
Locules number	LN	FR	QN	measured in 3 fruits of 2 plants per block = total measured fruits = 18
Ripening earliness	RE	FR	QN	Number of days from transplant until 50% of plants have at least one ripe fruit.
Ripening uniformity	RU	FR	QN	No. of days from transplant until all plants have at list one ripe fruit—No. of days from transplant until the first plant has a ripe fruit
Fruit weight	FW	FR	QN	Grams (g). Harvesting three fruits/plant on two plants/block for a total of 18 fruits per accession, and weight them individually
Total yield	TY	FR	QN	Grams (g). Measured from 1st to 6th trusses. Yield per plant = number of fruit set per plant × average weight of the fruit. Mean yield of one accession = average of yield of the 6 (2 per block) evaluated plants.
Fruits set truss	FST	FR	QN	Number (n°). Measured in two plants per block when truss 6 has set fruits by counting the number of commercial fruits set in each truss separately
Fruit set plant	FSP		QN	Total n° of fruits per plant considering all trusses
**Chemical traits**				
Soluble solids content	SS	FR	QN	° Brix degree. Evaluated in one fruit of two plants per block
pH	PH	FR	QN	pH scale. Evaluated in one fruit of two plants per block
Acidity	AC	FR	QN	Percentage (%). Evaluated in one fruit of two plants per block
**Root characterization**				
Radicular crown angle	RA	RT	QN	(°) Angle degree
Diameter of main root	DMR	RT	QN	Millimeters (mm) at the union with the plant stem
Density of fine roots	DR	RT	PQ	(Diameter ≤ 0.5 mm) index 1-5 (1 = very scarce, 2 = scarce, 3 = intermediate, 4 = abundant, 5 = very abundant)
Root weight*	RW	RT	QN	Grams (g)

^a^PL, Plant; FR, Fruit; RT, Root; ^b^PQ, Pseudo qualitative; QN, Quantitative; *Only at one location.

### Chemical Traits

A bulk of representative fruits for each accession/block/treatment were sampled and subsequently washed and dried. Soluble solid content was measured using 0.5 ml of liquid extract with digital refractometers (HI 96801, HANNA instruments, Padua, Italy; Refracto 30PX, Mettler-Toledo, Novate Milanese, Italy). The titratable acidity and pH were measured with a pH-Matic 23 analyzer titroprocessor equipped with a pH electrode including a temperature sensor (model 5011T) (Crison Instruments, Barcelona, Spain), using 10% (w/v) aqueous tomato extract and NaOH 0.1 M as titrating reagent. Titratable acidity was expressed as g citric acid/L juice.

### Root Architecture

The roots were assessed at the end of the cycle firstly removing soil and taking care not to damage the root system, then removing the substrate residues by carefully shaking the root. Two quantitative traits were measured including the radicular crown angle and diameter of the main root (Table 2). The density of fine roots (roots having diameters ≤ .5 mm) was scored using a scale from 1 (minimum value) to 5 (maximum value). The root weight was scored only in Italy by cutting the whole root apparatus at the collar junction.

### Data Analysis

The genotypic (σ^2^_G_) and phenotypic variances (σ^2^_P_ = σ^2^_G_ + σ^2^_E_ + σ^2^_T_ + σ^2^_G × E_ + σ^2^_G × T_ + σ^2^_G × E × T_) of each trait were obtained from the mean squares (MS) of the genotype, location, treatments, interactions, and residuals of the ANOVA performed, in order to estimate the broad-sense heritability (*H*^2^) using the formula $H^{2}=\sigma_{G}^{2}/\sigma_{P}^{2}$ (Wricke and Weber, 2010). The coefficients of genetic (GCV) and phenotypic (PCV) variation were estimated from the corresponding square roots of the variance components (σ_G_ and σ_P_) and the mean value of the trait (μ). The genetic advance as percent of population mean was also derived as GAM = [(σ_p_ × k × *H*^2^)/μ] × 100, where k is the selection intensity at 5% (2.06) (Falconer and Mackay, 1996).

A three-way ANOVA for determining genotype (G), location (E), and treatment (T) effects and the double and triple interactions was performed for every trait studied. In the ANOVA, treatments considered were the control (Etc100%, N 100%), WS (Etc30%, N100%), and NS (Etc100%, N0%). Mean square values (MS) were used to estimate the magnitude of the observed effect, while the total sum of squares in percentage (TSS%) was calculated dividing the TSS of the effect by the total TSS. The data from replicates per accession were used for removing the block effect. The mean values, standard deviation, coefficient of variation, and range for all traits were calculated from accession means and for the three treatments in both locations. Average differences between treatments and control were compared using the Dunnett tests; multiple group comparisons were performed using the Tukey range tests. A *p* = 0.05 threshold was considered to indicate a statistically significant difference. The traits with significant differences between treatments at each location were considered displaying a stable significant difference. All analyses were performed using the R statistical software v4.0.2.

The correlations among traits scored in the two locations and for each treatment were calculated from accession means using the *psych* and *corrplot* R packages. The Spearman linear coefficients of correlation (r) were calculated between pairs of traits and the significance of correlations was evaluated at *p* < 0.01. A principal component analysis (PCA) was carried out among accession means considering the three treatments and two locations in order to determine which are the most effective traits in discriminating among accessions. PCA loading and score plots were drawn using the computer package XLSTAT 2012.1. The prediction ellipses with a 95% level of confidence were added to the PCA score plot.

## Results

### General Statistics and Genetic Parameters

Considering locations and applied treatments, highly significant differences (*p* < 0.001) were found in the studied set for all traits except for disease in foliage and fruit that showed significance at *p* < 0.01 or no significance, respectively (Table 3). The greatest heritability in a broad sense (*H*^2^) was found for locules number (0.93), with fruit weight, fruit set truss, and fruit set plant displaying *H*^2^ values above 0.90. The lowest heritability values were instead found for the presence of pests and diseases, with the lowest *H*^2^ for disease in fruits (0.37). Among trait categories, root traits showed, in average, the lowest heritability. For all traits, the coefficient of PCV was from moderate to high, with values ranging from 11.37% (pH) to 161.71% (fruit set plants). The same trend was observed for the GCV. In general, the greatest GCV and PCV were found for agronomic traits, being in average 103.36 and 91.80, respectively. As expected, PCV values were higher than GCV in all instances, with a ratio PCV/GCV ranging from 1.02 to 1.36 for locules number and disease in fruits, respectively. Fruit features showed the highest genetic advance as a percentage of the population mean (GAM) with values above 100% for puffiness appearance, fruit fasciation, blossom end rot, locules number, fruit weight, fruit set truss, and fruit set per plant. On average, agronomic traits exhibited the highest GAM, while lower average values were instead observed for chemical traits. The lowest level (<12.04%) was observed for pH.

**TABLE 3:** Descriptive statistics for pseudo qualitative and quantitative traits, broad sense heritability (*H*^2^), phenotypic and genotypic coefficients of variation (PCV and GCV, respectively), and genetic advance as percent of population mean (GAM), for traits analyzed in three treatments (control, water stress, nitrogen stress) and two environments (Monsampolo del Tronto, Italy; Alcàsser, Spain) on 42 tomato cultivars.

Trait acronym^a^	R square	*F* ratio	*Prob* > *F*^b^	*H* ^2^	PCV	GCV	GAM
**Morphological plant and fruit descriptors**							
FD	0.39	2.93	***	0.59	40.42	34.90	31.92
PV	0.57	4.92	***	0.71	30.83	28.11	28.08
SP	0.53	4.47	***	0.69	73.87	66.77	65.87
FS	0.62	6.04	***	0.75	46.47	43.04	44.28
GS	0.77	11.28	***	0.85	80.10	76.77	84.01
PA	0.75	9.96	***	0.83	129.47	123.43	133.74
BE	0.18	1.65	***	0.45	39.94	31.52	25.16
FF	0.35	2.62	***	0.57	35.67	30.35	27.11
RC	0.71	8.39	***	0.81	90.56	85.61	91.34
**Agronomic traits**							
SD	0.30	2.30	***	0.53	34.92	29.16	25.31
RK	0.41	3.13	***	0.61	95.45	83.11	77.06
CC	0.34	2.55	***	0.56	115.18	97.64	86.76
FFA	0.66	6.76	***	0.77	130.78	122.06	127.29
BR	0.50	3.96	***	0.66	131.61	117.60	113.80
PF	0.12	1.41	***	0.41	65.17	49.88	38.09
PFr	0.20	1.75	***	0.47	96.45	76.93	62.33
DF	0.10	1.33	**	0.40	84.11	63.59	47.73
DFr	0.06	1.18	*ns*	0.37	99.77	73.46	53.15
LN	0.89	25.05	***	0.93	124.18	121.77	139.16
RE	0.76	10.69	***	0.84	25.05	23.95	26.11
RU	0.20	1.76	***	0.47	103.94	83.01	67.39
FW	0.88	23.19	***	0.92	146.45	143.39	163.38
TY	0.42	3.18	***	0.61	78.68	68.63	63.82
FST	0.86	19.54	***	0.91	160.33	156.38	176.88
FSP	0.87	22.08	***	0.92	161.71	158.16	179.86
**Chemical traits**							
SS	0.62	5.92	***	0.75	38.96	36.04	37.02
PH	0.79	12.23	***	0.86	11.37	10.93	12.04
AC	0.50	3.99	***	0.67	53.83	48.14	46.67
**Root characterization**							
RA	0.17	1.64	***	0.45	36.32	28.63	22.82
DMR	0.26	2.06	***	0.51	29.42	24.14	20.42
DR	0.35	2.67	***	0.57	60.57	51.66	46.40
RW	0.26	2.09	***	0.51	85.63	70.43	59.60

*^a^The full name of each trait abbreviation in the first column can be found in Table 2.*

^b^***, ** indicate significance at P < 0.001, P < 0.01, respectively; ns = not significant.

### Phenotypic Diversity in Response to N and Water Treatments

The range and mean values with the respective coefficient of variation for water and N stress and for the control in both environments are shown in Table 4. At each location, we found similar average levels for morphological traits without any differences among treatments except that for foliage density and blossom end scar conditions. On the contrary, higher variation was found for agronomic, chemical, and root characteristics between treatments and cultivation sites, although some exceptions were observed for fruit traits including radial cracking, fruit fasciation, locule number, ripening uniformity, fruit weight, and fruit set.

**TABLE 4:** Mean, coefficient of variation (in brackets) and results of *post hoc* tests for the traits measured in the selected set in two locations and control (CTRL), water stress (WS), and nitrogen stress (NS) treatments.

	CTRL ITALY		NS ITALY		WS ITALY		CTRL SPAIN		NS SPAIN		WS SPAIN	
	Mean/CV	Range	Mean/CV	Range	Mean/CV	Range	Mean/CV	Range	Mean/CV	Range	Mean/CV	Range
FD	4.57 (31.96) a	2.00–7.00	4.17 (32.45) a *	3.00–7.00	4.52 (33.32) a	1.00–7.00	5.09 (18.38) a	3.00–7.00	4.88 (16.83) ab	3.00–7.00	4.76 (18.04) b **	3.00–7.00
PV^	*na*	*na*	*na*	*na*	*na*	*na*	6.40 (17.66) a	3.00–9.00	6.19 (21.23) a	3.33–9.00	6.28 (18.71) a	4.00–9.00
SP	2.15 (44.11) a	1.00–4.00	2.09 (44.61) a	1.00–4.00	1.90 (46.34) a	1.00–4.00	1.44 (40.50) a	1.00–3.00	1.58 (38.82) a	1.00–4.00	1.47 (38.11) a	1.00–3.00
FS	5.55 (26.91) a	2.00–9.00	5.30 (26.78) a	2.00–9.00	5.44 (25.59) a	3.00–9.00	6.80 (27.42) a	3.00–9.00	6.59 (27.36) a	3.00–9.00	6.75 (24.90) a	3.67–9.00
GS	1.89 (44.10) a	0.00–3.00	1.80 (46.16) a	0.00–3.00	1.98 (41.32) a	0.00–3.00	1.76 (52.32) a	0.00–4.00	1.80 (49.97) a	0.00–4.00	1.77 (54.40) a	0.00–4.00
PA	1.54 (80.05) a	1.00–7.00	1.60 (79.84) a	1.00–7.00	1.42 (78.97) a	1.00–7.00	1.82 (81.45) a	1.00–7.00	1.83 (73.49) a	1.00–7.00	1.67 (71.58) a	1.00–7.00
BE	2.09 (31.85) a	1.00–3.00	1.92 (33.76) a	1.00–3.00	2.09 (28.09) a	1.00–3.00	2.08 (20.32) ab	1.00–3.00	2.22 (23.05) a **	1.00–3.00	2.04 (22.60) b *	1.00–3.00
FF	2.78 (27.45) a	1.00–4.00	2.89 (27.78) a	1.00–5.00	2.81 (27.82) a	1.00–4.00	2.89 (16.90) a	2.00–4.00	2.82 (18.18) a	2.00–4.00	2.86 (19.21) a	1.00–4.00
RC	2.57 (41.42) a	1.00–5.00	2.36 (42.80) a	1.00–5.00	2.41 (49.29) a	1.00–7.00	2.42 (62.64) a	1.00–7.00	2.28 (67.13) a	1.00–7.00	2.35 (63.01) a	1.00–7.00
SD	1.40 (21.63) a	0.60–2.10	1.35 (24.32) a	0.50–2.50	1.42 (21.65) a	0.80–2.20	1.52 (22.78) a	0.40–3.00	1.27 (18.93) b **	0.63–2.03	1.32 (23.77) b **	0.70–2.70
RK	2.98 (60.99) a	1.00–7.00	2.56 (64.44) a	1.00–7.00	2.58 (69.02) a	1.00–7.00	3.36 (51.04) a	1.00–7.00	3.62 (60.27) a	1.00–7.00	3.79 (54.79) a	1.00–7.00
CC	2.01 (76.91) a	1.00–7.00	1.96 (73.18) a	1.00–7.00	1.98 (77.08) a	1.00–7.00	2.20 (68.84) a	1.00–7.00	2.70 (70.47) ab	1.00–7.00	2.93 (72.20) b **	1.00–7.00
FFA	1.16 (47.21) a	1.00–3.00	1.10 (39.41) a	1.00–3.00	1.10 (39.41) a	1.00–3.00	1.67 (82.32) a	1.00–7.00	1.86 (79.33) a	1.00–7.00	2.06 (83.21) a	1.00–7.00
BR	1.33 (67.97) a	1.00–5.00	1.23 (63.28) a	1.00–5.00	1.50 (71.41) a	1.00–5.00	1.43 (73.04) b	1.00–6.33	1.68 (86.57) b	1.00–7.00	2.34 (84.44) a	1.00–7.00
PF	1.00 (0.00) a	1.00–1.00	1.00 (0.00) a	1.00–1.00	1.03 (24.60) a	1.00–3.00	1.26 (52.12) ab	1.00–3.00	1.18 (44.47) b	1.00–3.00	1.44 (56.54) a	1.00–7.00
PFr	1.52 (75.02) a	1.00–7.00	1.18 (48.61) b **	1.00–3.00	1.18 (53.24) b **	1.00–5.00	1.77 (56.15) a	1.00–5.00	1.77 (60.06) a	1.00–5.67	2.10 (60.68) a *	1.00–6.33
DF	2.68 (56.53) b	1.00–7.00	3.39 (44.47) a **	1.00–7.00	2.28 (53.91) b	1.00–5.00	3.17 (34.65) a	1.00–7.00	2.78 (79.49) ab	1.00–7.00	2.30 (66.84) b **	1.00–7.00
DFr	1.47 (62.94) a	1.00–5.00	1.34 (68.05) a	1.00–7.00	1.47 (71.92) a	1.00–7.00	1.11 (69.46) b	1.00–7.00	1.37 (69.23) a *	1.00–5.00	1.12 (69.47) ab	1.00–7.00
LN	5.09 (64.94) a	2.00–13.67	4.83 (67.21) a	2.00–14.33	5.01 (69.71) a	2.00–15.67	5.61 (77.06) a	2.00–18.83	5.56 (78.08) a	1.00–20.83	5.61 (75.39) a	2.00–17.17
RE	69.28 (16.34) a	44.00–98.00	70.07 (17.10) a	37.00–98.00	68.76 (14.74) a	35.00–93.00	65.03 (10.56) ab	49.00–79.00	66.57 (14.77) a	46.00–92.00	63.88 (13.21) b	37.00–88.00
RU	10.11 (75.70) a	0.00–41.00	10.09 (69.72) a	0.00–33.00	9.85 (71.07) a	0.00–32.00	7.26 (59.91) a	0.00–28.00	7.44 (49.12) a	1.00–24.00	6.42 (55.21) a	0.00–23.00
FW	133.20 (69.07) a	7.33–354.33	125.28 (78.85) a	4.00–399.50	124.60 (73.15) a	4.33–369.77	187.80 (90.30) a	6.79–805.00	176.70 (84.62) a	6.42–620.59	155.56 (91.08) a	6.74–757.69
TY	3,248.4 (53.01) a	187.50–10,773	2,668.4 (60.74) b **	148.50–8,827.0	2,957.9 (50.02) ab	287.00–9,269.0	3,519.3 (43.91) a	652.46–7,491.2	3,121.5 (46.17) ab	378.00–7,919.7	2,849.2 (46.79) b **	595.53–6,820.1
FST	6.18 (87.25) a	1.17–30.50	5.60 (92.91) a	0.67–24.67	6.30 (86.91) a	1.00–24.50	7.46 (97.52) a	0.92–34.83	6.30 (95.82) a	0.92–31.17	6.72 (100.91) a	1.08–39.75
FSP	37.07 (87.26) a	7.00–183.00	33.60 (92.92) a	4.00–148.00	37.81 (86.92) a	6.00–147.00	42.11 (101.10) a	5.50–209.00	37.82 (95.82) a	5.50–187.00	39.80 (102.06) a	6.50–238.50
SS	5.35 (23.22) b	2.60–9.70	5.09 (26.26) b	2.90–9.20	5.77 (23.98) a *	3.50–10.70	5.11 (22.88) b	3.10–9.80	5.17 (23.81) b	3.40–11.20	5.97 (19.88) a **	4.00–10.10
PH	4.19 (3.42) ab	3.88–4.73	4.22 (3.13) a	3.88–4.51	4.17 (3.10) b	3.79–4.49	4.73 (5.59) a	4.24–5.51	4.62 (5.25) b **	4.12–5.59	4.53 (4.78) c **	4.16–5.41
AC	0.68 (21.31) a	0.27–1.21	0.67 (35.64) a	0.42–2.62	0.72 (23.33) a	0.39–1.21	0.44 (31.63) b	0.20–0.88	0.46 (28.79) b	0.18–0.93	0.54 (29.17) a **	0.25–0.97
RA	123.58 (23.33) b	40.00–220.00	139.33 (23.77) a **	17.00–215.00	131.83 (22.24) ab	10.00–180.00	124.76 (21.88) a	70.00–190.00	104.05 (24.23) b **	50.00–180.00	131.55 (22.67) a	60.00–180.00
DMR	1.77 (15.94) a	1.20–2.80	1.67 (17.52) b *	1.00–2.50	1.69 (18.94) ab	0.40–2.80	1.74 (22.23) a	1.00–2.70	1.50 (17.47) c **	1.00–2.00	1.62 (20.40) b *	1.00–3.00
DR	2.66 (42.49) a	1.00–5.00	2.34 (42.65) a *	1.00–5.00	2.76 (36.20) a	1.00–5.00	3.20 (35.28) a	1.00–5.00	3.30 (34.11) a	1.00–5.00	3.49 (34.51) a	1.00–5.00
RW^	79.77 (50.60) a	19.10–237.50	78.80 (60.79) b	11.80–245.40	79.70 (59.14) a	12.00–262.80	*na*	*na*	*na*	*na*	*na*	*na*

*^Data available from single locations.*

*Significant different respect the control at p < 0.05; **significant different respect the control at p < 0.01.

*For traits within each location, means with different letters are significantly different (Tukey’s HSD, p = 0.05).*

*Means with asterisk are statistically significant in comparison to the respective control (Dunnett’s).*

*The full name of each trait abbreviation in the first column can be found in Table 2.*

Foliage density was higher in SP with respect to IT, although both showed intermediate values following the scale used. In both locations, a slightly greater density of foliage was found for the control with respect to the stress treatments, showing, in addition, significant differences between NS and the control in IT and WS and the control in SP (Table 4). This trend has been confirmed by plant vigor for the SP site which, although not significant among treatments, was on average higher and less variable in the control with respect to the two stress treatments. Among the other morphological traits, differences between locations for all treatments were found for style position and ribbing at the calyx end being higher in IT, whereas fruit set sequence and puffiness appearance were in average higher in SP. The remaining traits did not exhibit any substantial differences between locations except for the blossom end scar which showed an opposite trend under N stress between IT and SP with significant differences between treatments and control in the latter location.

Regarding agronomic traits, we found a bigger radial and concentric cracking in fruits harvested in SP, with values slightly higher in the WS and NS plots than those in the control (Table 4). In addition, concentric cracking significantly differed with respect to the control in the SP site. An opposite trend was found in IT, where both traits displayed higher values in the control condition. Overall, values encountered for both traits revealed a scarce fruit cracking for all cultivation conditions.

The fruit fasciation and blossom end rot were bigger in SP compared to IT for all treatments. In average, the blossom end rot values were higher under WS condition at both locations. Regarding pest and disease incidences, the plants were in good status in both fields with generally greater values only for disease in foliage. Except for pests in foliage, for which higher average values were found under WS conditions at both sites, different trends have been observed within each field for the other traits, thus highlighting how the reduction of water and N would not impact negatively on the general sanitary status of the fields. Interestingly, we observed that water reduction increased, although not significantly, ripening earliness and uniformity, whereas both water and N reduction led to a decrease in fruit weight and total yield. For the latter, significant differences in respect to the control were observed for NS in IT and WS in SP. Fruit set traits were more affected by a reduction in N, although no significant differences were found with control and water stress plots.

Among chemicals, soluble solid content significantly increased in both sites under water stress conditions, while the pH and acidity values were almost similar in the two locations with lower pH and consequent higher acidity encountered in the WS plots.

Finally, root traits were those differing most among treatments and locations. The radicular crown angle showed opposite trends, exhibiting the highest and lowest average values in IT and SP, respectively. Instead, at both locations, the diameter of the main root and the density of fine roots exhibited the best values under control and WS, respectively.

### Variance Component Analysis

The results of the factorial ANOVA for agronomic, chemical, and root traits under different stress treatments (T) in two experimental locations (E) are given in Table 5. A significant effect of the genotype (G) was found for all traits except for disease in fruits. In general, the main source of variation was due to the G, which accounted on average for 38.28% of the total variation, expressed by TSS%, ranging from 6.60 for disease in fruits to 84.08 for locules number. All traits were predominantly controlled by the genotype except for pH and acidity, for which the environment had a preponderant effect on their variation, explaining a TSS% of 53.01 and 26.08, respectively. Partitioning of TSS% in the other components affecting the variation highlighted a general smaller influence of T (average 1.63%) and E (average 5.65%), whereas, among the interactions between factors, G × E showed the highest average TSS% (8.54%).

**TABLE 5:** Multifactorial analysis and significant levels for genotype (G), environment (E), treatments (T), and combined effects (G × E, G × T, E × T, G × E × T) for pseudo qualitative and quantitative traits evaluated in this study on 42 tomato varieties grown under control, water stress (Etc30%, N100%), and nitrogen stress (Etc100%, N0) treatments in two locations (Italy and Spain).

Trait acronym	Genotype (G) *df = 41*		Treatment (T) *df = 2*		Locality (E) *df = 1*		G × T *df = 82*		G × E *df = 41*		T × E *df = 2*		G × T × E *df = 82*		Error *df = 504*
								
	TSS%	*F*	TSS%	*F*	TSS%	*F*	TSS%	*F*	TSS%	*F*	TSS%	*F*	TSS%	*F*	TSS%
**Morphological plant and fruit descriptors**															
FD	36.50	10.91***	1.04	6.40*	4.19	51.33***	4.03	0.60^NS^	7.41	2.27***	0.58	3.56*	5.60	0.86^NS^	40.65
SP	41.62	16.20***	0.60	4.79*	10.71	170.88*	3.86	0.75^NS^	6.86	2.74*	0.51	4.09*	4.64	0.92^NS^	31.20
FSs	45.30	21.23***	0.29	2.80^NS^	12.44	239.09***	3.67	0.86^NS^	9.69	4.90***	0.00	0.03^NS^	3.31	0.837^NS^	25.29
GS	70.34	56.41***	0.14	2.36^NS^	0.15	5.09*	2.00	0.80^NS^	9.60	7.89***	0.25	4.06*	2.38	0.98^NS^	15.14
PA	69.09	50.08***	0.31	4.63*	1.11	33.02***	2.79	1.01^NS^	6.89	5.12***	0.01	0.11^NS^	3.05	1.13^NS^	16.76
BE	15.72	3.48***	0.03	0.13^NS^	0.54	4.93*	9.32	1.03NS	8.21	1.86*	1.98	9.01***	9.36	1.06^NS^	54.83
FF	32.78	9.17***	0.01	0.07^NS^	0.04	0.45^NS^	6.64	0.93^NS^	10.88	3.12***	0.33	1.8^7NS^	5.91	0.85^NS^	43.40
RC	63.64	40.01***	0.30	3.86*	0.07	1.78^NS^	2.67	0.84^NS^	11.11	7.16***	0.02	0.23^NS^	2.87	0.93^NS^	19.32
**Agronomic traits**															
SD	26.96	7.02***	3.61	19.27***	0.09	0.99^NS^	6.75	0.88^NS^	6.53	1.74**	2.57	13.72***	6.83	0.91^NS^	46.66
RK	30.03	9.39***	0.05	0.34^NS^	5.76	73.85***	6.52	1.02^NS^	10.61	3.40***	0.85	5.46**	7.34	1.18^NS^	38.83
CC	27.20	7.53***	0.69	3.90*	3.66	41.56***	9.65	1.34*	5.58	1.58*	0.90	5.12**	8.45	1.20^NS^	43.87
FFA	30.88	16.44***	0.30	3.26*	10.06	219.69***	3.05	0.81^NS^	30.23	16.50***	0.62	6.72**	2.06	0.56^NS^	22.81
BR	34.16	12.35***	3.23	23.95***	3.31	49.12***	8.51	1.54**	8.53	3.16***	1.30	9.64***	7.38	1.37*	33.58
PF	8.72	1.81**	1.29	5.50**	7.6	64.50**	7.41	0.77^NS^	8.85	1.88**	0.95	4.03*	6.53	0.69^NS^	58.66
PFr	8.73	1.99***	0.57	2.69^NS^	8.31	77.53***	10.70	1.22^NS^	5.90	1.38^NS^	1.63	7.60***	10.76	1.25^NS^	53.39
DF	9.99	2.03***	4.57	19.00***	0.01	0.01^NS^	7.98	0.81^NS^	8.09	1.68**	2.17	9.01***	7.27	0.76^NS^	59.92
DFr	6.60	1.27^NS^	0.10	0.41^NS^	1.56	12.38***	7.25	0.70^NS^	12.03	2.38***	0.90	3.55*	8.61	0.85^NS^	62.94
LN	84.08	137.87***	0.03	0.98^NS^	0.81	54.79***	1.17	0.96^NS^	5.22	8.78***	0.01	0.41^NS^	1.27	1.06^NS^	7.41
RE	65.28	49.82***	3.73	116.74***	0.65	10.22***	7.04	5.51***	4.35	1.66***	0.08	1.28^NS^	2.95	1.15^NS^	15.92
RU	12.78	2.91***	6.14	57.34***	0.22	1.05^NS^	11.83	2.76***	6.89	0.78^NS^	0.08	0.35^NS^	8.74	1.02^NS^	53.33
FW	75.95	116.33***	0.40	12.63***	3.56	223.82***	1.97	1.51**	7.82	12.28***	0.17	5.32**	2.21	1.73***	7.93
TY	39.38	12.36***	2.19	14.10***	0.43	5.55*	5.68	0.89^NS^	6.57	2.11***	0.62	4.00*	6.44	1.04^NS^	38.69
FST	81.28	105.57***	0.38	10.07***	0.04	2.28^NS^	1.69	1.10^NS^	5.48	7.30***	0.09	2.29^NS^	1.69	1.12^NS^	9.35
FSP	83.80	121.83***	0.24	7.19***	0.01	0.23^NS^	1.21	0.88^NS^	5.13	7.64***	0.04	1.21NS	1.22	0.91^NS^	8.36
**Chemical traits**															
SS	52.43	25.12***	6.21	60.98***	0.01	0.13^NS^	3.88	0.93^NS^	6.78	3.33***	0.52	5.09**	4.87	1.20^NS^	25.30
PH	16.57	14.13***	2.35	41.14***	53.01	1,854.20***	3.62	1.54**	5.85	5.12***	1.62	28.32***	2.77	1.21^NS^	14.21
AC	16.18	5.81***	2.51	18.45***	26.08	383.78***	5.17	0.93^NS^	10.65	3.92***	0.47	3.48*	5.17	0.95^NS^	33.77
**Root characterization**															
RA	11.23	2.46***	1.91	8.58***	3.12	28.07***	8.23	0.90^NS^	5.41	1.22^NS^	7.34	33.05***	7.54	0.85^NS^	55.23
DMR	22.86	5.60***	4.43	22.29***	1.72	17.30***	5.69	0.70^NS^	8.13	2.04*	0.74	3.69*	6.98	0.88^NS^	49.45
DR	28.35	8.01***	1.15	6.67**	10.19	118.09***	4.96	0.70^NS^	5.08	1.47*	0.56	3.24*	6.83	0.99*^NS^*	42.88

*, **, ***, significant at P < 0.05, P < 0.01, P < 0.001, respectively;

*^NS^ not significant. df, degrees of freedom; TSS, total sum of squares; F, F ratio.*

A highly significant variation due to T was found for 14 traits including most agronomic quantitative traits, chemicals, and root traits, except for the density of fine roots (Table 5). On the contrary, the morphological traits showed a low level of significance (*p* < 0.05). The effect of E was statistically significant for all categories of traits, except for fruit firmness, ribbing at calyx end, stem diameter, disease in foliage, ripening uniformity, fruit set truss and plant, and soluble solids.

Most interactions among factors were found between G and E for which highly significant differences (*p* < 0.001) have been detected for 19 out of the 30 traits scored at both locations. Instead, for G × T and T × E, only 2 and 7 out of the total 30 traits, respectively, exhibited significant values. A variable degree of significant interactions was found for the environmental × treatment effects in several traits. Overall, only the fruit weight (*p* < 0.001) and the blossom end rot (*p* < 0.05) showed high level of significance for the interaction among the three factors (G × E × T).

### Quantitative Trait Performance of Varietal Types

**FIGURE 2:**
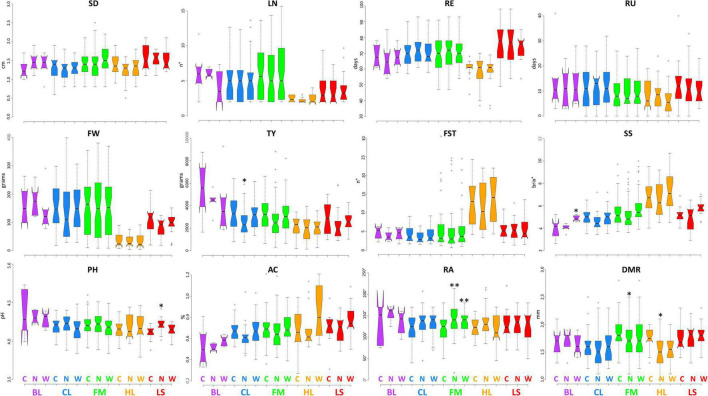
Variation for quantitative traits in the five cultivar groups grown under control and stress conditions in Italy. Notched boxplots showing median values and quartiles for the five considered cultivar groups grown in Italy under control (C), water deficit (W), and nitrogen stress (N). The measurement scale for each trait is reported on the Y-axis; details in Table 2. The asterisks indicate significant differences with respect to the control condition according to the Dunnett’s test; the single (*) and the double (^**^) asterisk indicates *p* < 0.05 and *p* < 0.01, respectively. BL = breeding lines, CL = elite cultivars, FM = landraces for the fresh market, HL = heirlooms, LS = long shelf-life accessions.

**FIGURE 3:**
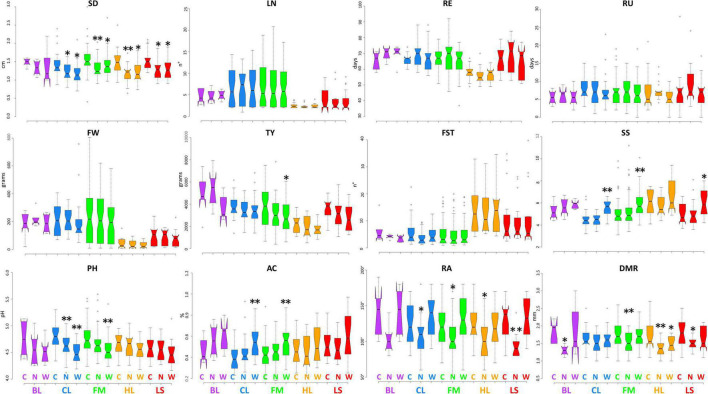
Variation for quantitative traits in the five cultivar groups grown under control and stress conditions in Spain. Notched boxplots showing median values and quartiles for the five considered cultivar groups grown in Spain under control (C), water deficit (W), and nitrogen stress (N). The measurement scale for each trait is reported on the Y-axis; details in Table 2. The asterisks indicate significant differences with respect to the control condition according to the Dunnett’s test; the single (*) and the double (^**^) asterisk indicates *p* < 0.05 and *p* < 0.01, respectively. BL = breeding lines, CL = elite cultivars, FM = landraces for the fresh market, HL = heirlooms, LS = long shelf-life accessions.

Figures 2, 3 display the performance of different varietal types for the quantitative traits tested in IT and SP, respectively. We observed a general reduction of stem diameter under WS and NS trials in the SP site with significant differences with respect to the control in all cultivar groups except for BL, whereas, in IT, the reduction has been observed only for CL and HL. The diverse number of locules was instead more evident between cultivar groups, while no substantial differences were found among treatments except for the BL at the IT site. In both locations, HL cultivars had an earlier maturation while LS accessions showed an opposite trend, more marked in Spain. Greater variation was found for the characteristics related to productivity and quality. A higher fruit weight was observed in both locations for BL, CL, and FM cultivars, with a more evident difference between LS and HL in Italy. For all cultivar groups, a higher yield was detected in SP. The breeding lines which include two hybrids developed by CREA showed the highest values at both sites, whereas HL had the lowest productivity, although exhibiting generally higher values of fruit set. While in IT, the lowest yield has been found under low N, in SP, water stress had a greater impact on the production. Soluble solids were instead higher in the HL with respect to the other cultivars, although outstanding values have been also detected for FM. In these two groups, we found accessions showing over 10 °Brix in both locations. All cultivar groups showed an increase of soluble solids under water stress compared to the other conditions, with significant differences with respect to the control observed for BL in IT, and for CL, FM, and LS in SP. Under N stress, in IT, we found a general decrease in soluble solids in all varietal groups compared to the control, whereas in SP, except for HL, similar or higher values were observed. A different trend for pH and acidity values was found across locations. In SP, a consistent decrease of pH and consequent increase of acidity were observed in all cultivar groups when either N reduction or water stress was applied. In IT instead, the N stress led to lower acidity of fruits. Differences were also found for root traits between the varietal groups in the two sites, being more evident in SP where a consistent reduction of radicular crown angle and diameter of the main root has been observed under N reduction. For the latter trait, in both sites, higher values were found for LS and FM.

### Best-Performing Accessions for Yield- and Quality-Related Traits

**FIGURE 4:**
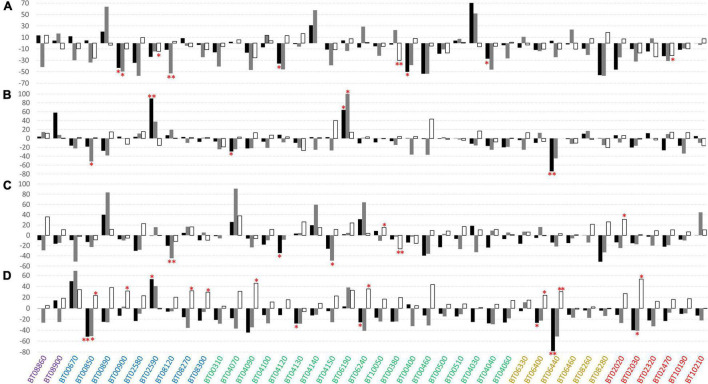
Phenotypic differences between water and nitrogen stress conditions compared to the control for the 42 tomato varieties tested in two locations. Values are expressed as differences in percentage between the mean of the stress condition with respect to the control. Different colored bars indicate different traits: fruit weight in black, total yield in gray, and soluble solids in white. **(A)** Nitrogen stress condition in Italy, **(B)** nitrogen stress condition in Spain, **(C)** water stress condition in Italy, and **(D)** water stress condition in Spain. The color of the accessions indicates the related cultivar type as follows: breeding lines (BL) in purple; elite cultivars (CL) in blue, landraces for the fresh market (FM) in green, heirlooms (HL) in dark orange, long shelf-life landraces (LS) in red. Single and double asterisks indicate significant differences with the control at *p* < 0.05 and *p* < 0.01, respectively.

The mean values, standard deviation, and results of *post hoc* tests for the traits analyzed under diverse levels of water and N supply in the 42 accessions are reported in Supplementary Table 2 for IT and Supplementary Table 3 for SP. Considering the yield, fruit weight, and soluble solids as the best predictors of the performance of cultivars, the phenotypic difference expressed as a percentage of the treatments compared to the control was calculated for each accession (Figure 4). We found a general reduction of the fruit weight once a stress was applied, although several accessions showed better performance at least in one of the independent trials. BT06190 (FM) was the most promising variety exhibiting an increase of fruit weight in both IT and SP under NS (Figures 4A,B) and WS (Figures 4C,D) conditions. For this accession, we found a significant increase of fruit weight in SP under N stress (272.48 vs. 165.81 g of the control). Except for the heirlooms, at least one accession for each varietal group showed an increase in fruit weight in both locations for the stresses applied. The increases in fruit weight were principally related to the locality rather than to the type of applied stress, showing in several cases discordant results between IT and SP. However, BT04030 (FM) and BT02590 (CL) showed the greatest increase of average fruit weight under N stress, with a +70% (366.28 g of NS vs. 215.22 g of the control) and +90% (180.68 g of NS vs. 95.16 g of the control) in IT and SP, respectively.

As for the total yield, the impact of the stresses applied was greater with respect to what was observed for fruit weight, in fact, non-accession showed an increase of yield across all stress treatments, and only one BT06240 (FM) showed an increase of yield in 3 out of the 4 trials. In most cases, we found an opposite trend in yield for the same type of stress in the two grown sites, with more consistent increase and decrease effects in IT. A remarkable increase of yield has been found for BT00890 (CL) for NS (+64%) and WS (+84%) in IT (Figures 4A,C and Supplementary Table 2), BT04140 (FM) (+58%) and BT04030 (+52%) under NS in IT (Figure 4A), and BT06190 (+102%) under NS in SP (Figure 4B and Supplementary Table 3). For water stress, BT04070 (FM) and BT02580 (CL) were the most promising accessions in IT and SP, exhibiting an increase of yield of +91 and +68%, respectively. Overall, within elite cultivars and landraces for the fresh market, it was possible to select genotypes showing good performances under stress conditions.

Contrary to the yield-related characteristics, the reduction of cultivation inputs has determined an increase of soluble solids in different accessions, with very evident effects under water stress (Figures 4C,D). Indeed, for the latter, almost all accessions including the entire set of BL and LS cultivars showed an increase of soluble solids in both locations. In all varietal groups, we found specific genotypes increasing °Brix degree in all trials. Among the BL, BT08860 increased °Brix up to +36% under WS in IT, whereas within the elite cultivars, BT02580 increased soluble solids of +34% under WS in SP. Within landraces for the fresh market, it was possible to identify several promising accessions, and among these, BT04090 reached values up to +46% under WS in SP. For long shelf-life accessions, BT02020 was found to be very promising with an increase of soluble solids of +31% and + 27% in IT and SP under WS, respectively. Finally, among heirlooms, BT06400 and BT06440 showed a statistically significant increase of soluble solids over the control under WS in SP, although the trend was not confirmed in the other stress treatments.

### Principal Component Analysis and Correlation Among Traits

**FIGURE 5:**
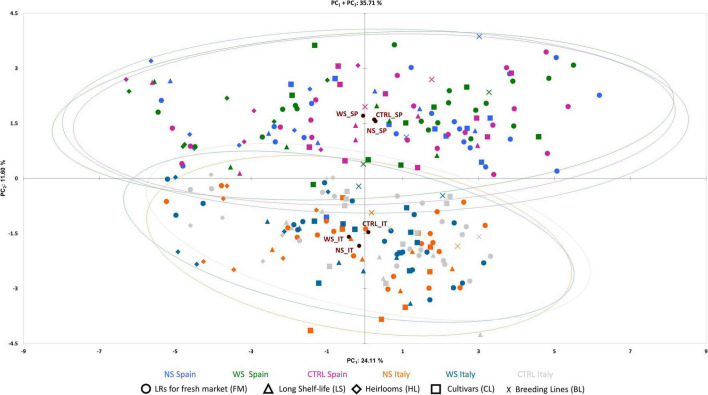
Principal component analysis. Loading plot of the first (PC_1_) and second (PC_2_) principal components showing the variation for 30 traits scored in two environments. Accessions of different cultivar groups are represented by different colored symbols according to the applied treatment and cultivation site. Color and symbols are listed below the graph. Ellipses grouped the accessions for each treatment with a 95% CI.

**FIGURE 6:**
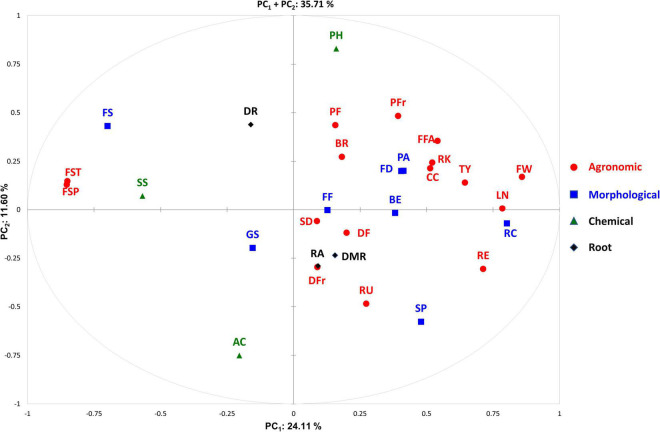
Distribution of the traits scored on the PCA biplot. The direction and distance from the center of the biplot indicate how each OTU contributes to the first two components. The different category of traits is indicated using different color codes. Trait acronyms are listed in Table 2.

The PCA in the first two dimensions explained 35.71% of the total variation observed, with the first (PC_1_) and the second (PC_2_) components accounting for 24.1 and 11.60% of the total variation, respectively (Figure 5). PC_1_ was positively correlated with 23 out of the 30 traits scored in two locations excluding density of fine roots, green shoulder, acidity, soluble solids, and fruit set traits (Figure 6). PC_2_ was instead positively correlated with seventeen traits. The fruit weight and fruit set plant were the main factors discriminating the genotypes under study in the first component accounting for 10.21 and 10.05% of the total variation, respectively (Supplementary Table 4). pH and acidity explained 19.73 and 16.19% of the total variation of the second component, respectively. The PCA clearly separated accessions grown in SP and IT, being the former distributed in the positive axis of the PC_2_ while the latter clustered in the negative part. Regardless of the location, the accessions were evenly distributed in both the negative and positive axes of the PC_1_. The PCA did not clearly distinguish the control trials from WS and NS ones, although the barycenter of the confidence ellipses at both cultivation sites followed the same trend for the WS and control conditions being in the negative and positive axes of PC_1_, respectively.

**FIGURE 7:**
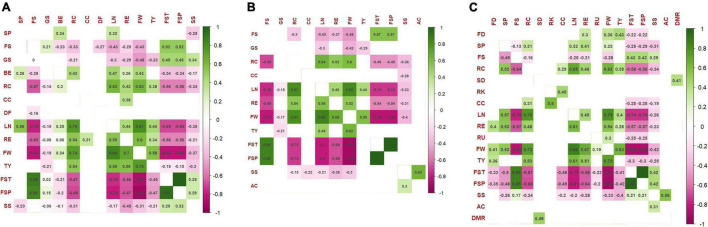
The Spearman’s rank significant correlations between pairs of traits evaluated in 42 tomato accessions. **(A)** Control, **(B)** nitrogen stress, and **(C)** water stress. Correlation coefficients are indicated in each cell. Colored cells are those with *p*-value < 0.01. Color intensity is directly proportional to the coefficients. According to the scale on the right, green and purple colors correspond to positive and negative correlations, respectively. Correlations among traits in IT are shown below the diagonal; correlations in SP are above the diagonal. The full name of each trait abbreviation can be found in Table 2.

The correlation among traits has been calculated for each treatment considering a significance threshold *p* < 0.01 using the Spearman coefficient. The correlogram within control condition is reported in Figure 7A. At both sites, ribbing at calyx end was positively correlated with several agronomic traits showing stronger *R* values for fruit weight and locule number. Negative correlations were instead found with fruit set traits. On the opposite, fruit set traits exhibited negative correlations with agronomic traits, the strongest ones found for fruit set truss and fruit set plant with locule number (*r* > −0.75 in SP and *r* = −0.69 in IT) and fruit weight (*r* > −0.87 in SP and *r* = −0.82 in IT). Positive correlations were detected between fruit weight and locule number (*r* > 0.8 in both locations), ripening earliness (*r* > 0.6 in both locations), and total yield (*r* = 0.74 in SP and *r* = 0.58 in IT). No correlations were detected for root traits, whereas, among chemicals, only soluble solids showed negative correlations with different agronomic traits although with a coefficient of less than 0.50 in all instances. Regarding the NS treatment, we found fewer significant correlations than for the control, whereas those confirmed showed higher coefficients (Figure 7B). More robust correlations with respect to the control were indeed found for fruit set traits, locule number, fruit weight, and fruit sequence. In addition, acidity and soluble solids were positive correlated at both sites with higher values in SP. The greatest number of correlations were observed within the WS fields (Figure 7C). The correlations already detected in the NS, and control fields were confirmed, being in several instances more robust than the control condition. In addition, several new significant correlations were observed between morphological and agronomic traits, as well as between the diameter of the main root and the stem diameter.

## Discussion

This study represents a step forward toward the identification of resilient cultivars adapted to low-input conditions. While investigations highlighting the potentiality and constraints of conventionally and organically cultivated tomatoes have already been performed (Barrett et al., 2007; Mitchell et al., 2007; Juroszek et al., 2009; Hallmann, 2012), fewer attempts have been so far focused on different cultivation methods in organic farms. The sought-after goal is to identify the best cultivars in organic farming and select those that are well suited by providing additional stresses. To that end, we explore the environmental factors underlying the variation of traits and the genotypic performance in 42 tomato accessions, mostly landraces and/or neglected materials, in trials across different organic farming environments. We focused on organic cultivation practices being a valid alternative to conventional ones for cultivating tomatoes under low-input conditions and with an increasing demand. To the best of our knowledge, this is the first attempt to study the genotypic and environmental effects in a tomato diversity panel grown under organic farming applying both water- and N-stress conditions. Organic farming relies on a series of management strategies aiming to reduce the impact of chemicals, ensuring the health of the ecosystem and biodiversity (Morshedi et al., 2017; Le Campion et al., 2020). The increasing awareness of food safety has enlarged the choice and consumption of products obtained with organic cultivation systems (Lazaroiu et al., 2019). However, to successfully achieve these objectives, it is essential to develop organically adapted cultivars. It has been widely discussed the urgent need for the selection of tomato adapted to organic farming considering that most of the varieties released annually are specifically bred for high-input conditions and target traits (e.g., yield, resistances) are not fully expressing their potential under low-input conditions (Lammerts van Bueren et al., 2011). Thus, developing such varieties requires specific testing in those stress conditions.

### Trait Variation Under N and Water Deficits

For the traits assayed, we found high phenotypic variability in the collection studied, which provides a potential source to exploit for selecting and breeding new varieties for the organic sector. For most traits, at both sites, the control condition showed higher values, whereas the coefficient of variation was lower either in WS or NS, highlighting less variation when the stress was applied. Although morphological traits did not substantially vary between treatments, the observed general decrease of foliage density, plant vigor, and stem diameter in one or both stress trials highlighted how the reduction of water and N affects the vegetative plant development. This could be explained by both minor photosynthetic activity and diminished carbon assimilation occurring with N and water deficit that leads to reduced biosynthesis of major plant macromolecules (Hou et al., 2020). In most accessions, we did not observe changes in puffiness appearance and fruit firmness, suggesting that the input shortages do not impact fruit texture. Previous studies report an increase of fruit firmness under water stress due to a lower transpiration rate, which combined with a reduced cuticle permeability decreases water loss and strengthens cell walls (Guichard et al., 2001; Barbagallo et al., 2012; Romero and Rose, 2019). However, other reports showed that the deficit of irrigation did not significantly impact fruit firmness (Zheng et al., 2013; Van de Wal et al., 2017; Zhang et al., 2017). The contrasting results could be due not only to the different plant materials used, which in our case included heterogeneous varietal types, but also to the type of water deficit applied (Van de Wal et al., 2017).

Watering, in synergy with other physiological, agronomic, and genotypic factors, is responsible for blossom end rot in tomato fruits (Hagassou et al., 2019) and increased plant disease (Obreza et al., 1996). An adequate consistency affects the shelf life and the general health of the fruits by reducing mechanical damages (e.g., cracks) and pest infection. The observed levels of cracking and disease in fruits under NS and WS suggest that the tomato panel studied responds well to low-input growing conditions.

For quantitative agronomic traits, we observed minor performances of the studied accessions when grown under stress conditions. The application of WS and NS had different effects on fruit ripening. While N reduction delayed fruit maturation in most cultivars and at both locations, water deficit had the opposite effect. Previous studies highlighted a shortened ripening period when water stress is applied, reporting an increase in vitamin C and carotenoids when the deficit is applied during the maturation stage (Ripoll et al., 2016; Medyouni et al., 2021). In addition, it has been observed that low N content delays fruit development, reducing furthermore the content of sugars, thus impacting negatively the overall quality (Khan et al., 2015). The decrease in yield and fruit weight under reduced irrigation was compensated by higher content of soluble solids. So far, many authors have found increases in the content of soluble solids when water scarcity is imposed (Johnstone et al., 2005; Barbagallo et al., 2012; Zhang et al., 2017; Lu et al., 2019). The increase is linked to different factors, including varietal choice and the magnitude of applied water deficit (Lu et al., 2019). Soluble solids mostly comprise carbohydrates and organic acids, which are precursors of the flavor and taste of fruits (Salles et al., 2003). Considering that in different cultivars the effects of water stress were not significantly different compared to the control, the optimal combination of water reduction in specific phenological phases of the plant cycle could lead to an improvement in the nutritional quality of fruits. N deficit had a remarkable effect on the reduction of total yield, highlighting how deprivation of N fertilization under organic conditions compromises tomato productivity. It must be considered that a drastic N reduction has been applied in the NS trials, suggesting that a mild reduction of N fertilization can be applied without affecting the yield performance. Rosa-Martínez et al. (2021) reported no yield reduction in long shelf-life tomatoes when N is reduced to 60% of the level commonly provided in the cultivation. This is likely due to the natural supply of N by the soil linked to organic agronomic practices that surely lead to better sustainable cultivations.

We also explored the root architecture, as the main system of water and nutrient uptake from the soil focusing on anatomical changes of main and fine roots. In most accessions, we found a higher radical angle and a greater density of fine roots under water-stress conditions. These results highlight how tomato roots tend to cope with stress conditions, expanding into the soil by increasing the angle and the number of fine roots. Similar results have been observed in winter wheat, where the increase of root density was observed by reducing the amount of water supplied compared to the control (Fang et al., 2017). Furthermore, the smaller diameter of the main root under water stress could be linked precisely to a root elongation mechanism at the expense of thickening. Although measured in a single environment, root weight did not change either under WS and NS, suggesting that stresses impacted only the root anatomy. This agrees with Hernandez-Espinoza and Barrios-Masias (2020) reporting similar biomass between dry and wet treatments in tomato.

### Genotypic and Environmental Effects on the Expression of Traits

Despite the level of diversity observed in the 42 studied accessions, we found that the environment had a major influence, compared to stress treatments, on the variation of traits. This is highlighted in the PCA that shows, in the bi-dimensional plot, the clear separation of accessions cultivated in the two locations. Only for a few traits, the variation due to treatments was predominant with respect to the environment. Among these, ripening uniformity, although exhibiting small genotypic influence, did not show any effect associated with the different cultivation sites and to its interaction with G and T. The high heritability coupled with low variation due to E and T interaction highlighted those traits having a strong genotypic control. In particular, the values observed for E, T, and their interactions for green shoulder, ribbing at calyx end, and locules number highlighted the possibility to maintain the main fruit features regardless of growing conditions. In addition, fruit weight and number of fruits per plant showed a high heritability, which, in combination with a high genetic advance (GAM), suggests additive gene action and an enhanced expression in offspring (Falconer and Mackay, 1996). On the opposite, traits exhibiting both low heritability and low GAM, such as root architecture and disease-related traits, are more subjected to the environmental and stress-treatment effects being more difficult to control during selection. The low number of traits showing no significant G × T and G × T × E highlights how the treatment effect minimally affects the total variation, suggesting the possibility to perform cultivar selection either under water stress and/or N reduction.

Better yield-related performances were evidenced at the SP location, suggesting pedo-climatic conditions more suitable for the cultivation of the tomato set studied. The most evident differences between locations were the clay-loam composition and the greater organic matter content of the SP soil. These properties would confer an intermediate compaction degree able to increase the available water capacity and aeration, enhancing furthermore the efficiency of roots for nutrient uptake, in particular the N stored in organic form (Nicolás et al., 2019; Soinne et al., 2020). In addition, the SP soil had a lower electrical conductivity resulting in a low level of salinity and higher N content than IT, thus conferring better conditions for plant growth (Chen et al., 2005).

We found the variation of pH and acidity was highly influenced by environmental effects rather than water and N stress. Contrariwise, the soluble solid content had a stronger genotypic control, being more influenced by the variability due to treatment, and less by that due to cultivation site. This highlights the complexity of breeding for taste-related traits, thus suggesting a different difficulty level of achievement in the improvement objectives for sourness and sweetness.

The deficit of water has strengthened the correlations among traits, highlighting the complexity of the physiological mechanisms of adaptation in response to drought (Osakabe et al., 2014), suggesting furthermore a greater synergy between the hypogeal and epigeal traits when water stress is applied. The approach pursued provides relevant information to discern the phenotypic performances and the interactions between G, E, and T. By estimating heritability, GAM values, and the robust correlations found in both locations and stress trials, we provide new hints on the strategies to adopt for breeding and selection, in order to achieve the best performance of cultivars in response to different environmental and cultivation conditions.

### Breeding Cultivars for Organic Farming

The tomato set examined in this study is part of a larger collection developed in the frame of the H2020 EU project BRESOV (Breeding for Resilient, Efficient and Sustainable Organic Vegetable production) which aims at improving the competitiveness of crops in organic and sustainable environment. We investigated 40 accessions selected based on the information retrieved from high-density genotyping (Esposito et al., 2020) and large-scale phenotyping (Tripodi et al., 2021) of over 240 diverse tomato cultivars. Two additional hybrids were included. Different cultivar groups were considered, given their morphological diversity, qualitative properties, and degree of appreciation by consumers. Among these, the “de penjar” and “da serbo” types being characterized by small fruit size and long shelf-life are suitable for cultivation in marginal environments often characterized by drought conditions (Figàs et al., 2018). The other groups were mostly represented by landraces, heirlooms, and elite cultivars comprising materials potentially suitable for organic farming cultivation and/or already diffused in local markets (Migliori et al., 2012). We also evaluated two novel hybrids that had a very good performance under low-input conditions. Evaluating these diverse materials in the two sites under the three conditions allowed the identification of candidates with good performance and resilience. The comparison of the performances of individual accessions sheds light on the possibility to exploit the hybrid vigor as a strategy to improve the yield-related traits and the root architecture in organic cultivation, as observed for BT08860. This agrees with previous findings in wheat (Vijaya Bhaskar et al., 2019) and maize (Burger et al., 2008), highlighting hybrids as very promising for organic farming providing increasing yield and greater vigor in the development of roots and shoots. Therefore, additional investigations are recommended in tomato to determine the effective use of hybrids for organic cropping systems.

Among traditional varieties such as landraces and heirlooms, genotypes with high-soluble solid content have been detected, highlighting their potential for further exploitation of quality traits. Overall, individual accessions improving trait performances under either N- or water-stress conditions were also identified in each cultivar group; among elite cultivars, BT00900 and BT08270 showed good performances in terms of yield, soluble solid content, and fruit set per plant under stress conditions, whereas several landraces for the fresh market (e.g., BT04140, BT04070), heirlooms (e.g., BT06400), and long shelf-life genotypes (e.g., BT10190, BT10210) were highly promising for several agronomic traits in one or both locations. This highlights how there are different genotypes that can be used for organic farming, allowing the reduction of cultivation inputs, but also how new breeding programs can be established for combining traits of interest and exploiting heterosis.

## Conclusion

In this study, we evaluated 42 tomato accessions in two organic farms located in typical Mediterranean environments in Italy and Spain by applying diverse cultivation management conditions based on the reduction of water and N supply. To broaden the selection of promising cultivars for low-input growing conditions, we considered a diversified panel including different groups of accession for specific consumer/market segments. We found how the epigeal part of the plant was minor influenced by the stress applied with respect to the hypogeal one, confirming how the root architecture plays a major role in plant adaptation to nutrient and drought stress. Water stress was instead more responsible for physiological malformations such as blossom end rot and fruit fasciation, while the general sanitary status of the fields linked to the occurrence of pests and disease was almost unchanged between the control and the stress trials. Beyond the wide diversity found for the traits assayed, we observed that a reduction in water and N fertilizer can be applied without affecting dramatically the overall performance. The results highlighted several accessions with either better production or soluble solid content in the stress trials, thus suggesting the possibility to improve the organoleptic quality by maintaining, furthermore, adequate yields. The partitioning of the G, E, and T effects and their interactions suggested the possibility to select broadly adapted genotypes able to maintain main morpho-agronomic characteristics in diverse cultivation environments and/or following different agricultural practices, shedding light on the possibility to promote different cultivars for organic farming, as well as establishing new cross-breeding programs. In addition, the comparison of the performances of individual accessions highlights the possibility to exploit the hybrid vigor as a strategy to improve yield-related traits and root architecture in organic cultivation; indeed, among accessions essayed, the hybrids had an optimal performance for most of the traits. The results of this work represent a step forward toward the selection of promising cultivars for low-input growing conditions, being also the first effort toward the assessment of both drought and N stress under organic cultivation. The established panel of tomato accessions provides a framework for further investigations on nutritional and sensorial properties, providing novel information to enhance the consumers’ preference for organically developed products.

## Data Availability Statement

The original contributions presented in this study are included in the article/Supplementary Material, further inquiries can be directed to the corresponding author/s.

## Author Contributions

PT, JP, TC, and MD conceived the project and coordinated the experiments. GC, FL, SS, and MF designed the experimental layout, managed the field trials, and performed the phenotypic characterization. FL and SS collected and curated the data. PT analyzed the data and drafted the manuscript. All authors critically revised the manuscript and approved the submitted version.

## Conflict of Interest

The authors declare that the research was conducted in the absence of any commercial or financial relationships that could be construed as a potential conflict of interest.

## Publisher’s Note

All claims expressed in this article are solely those of the authors and do not necessarily represent those of their affiliated organizations, or those of the publisher, the editors and the reviewers. Any product that may be evaluated in this article, or claim that may be made by its manufacturer, is not guaranteed or endorsed by the publisher.
